# Depth in arrangements: Dehn–Sommerville–Euler relations with applications

**DOI:** 10.1007/s41468-024-00173-w

**Published:** 2024-05-03

**Authors:** Ranita Biswas, Sebastiano Cultrera di Montesano, Herbert Edelsbrunner, Morteza Saghafian

**Affiliations:** IST Austria (Institute of Science and Technology Austria), Klosterneuburg, Austria

**Keywords:** Arrangements of great-spheres, Euler characteristics, Dehn–Sommerville relations, Discrete Morse theory, Neighborly polytopes, Counting, Theory of computation, Computational geometry

## Abstract

The depth of a cell in an arrangement of *n* (non-vertical) great-spheres in ${\mathbb {S}}^d$ is the number of great-spheres that pass above the cell. We prove Euler-type relations, which imply extensions of the classic Dehn–Sommerville relations for convex polytopes to sublevel sets of the depth function, and we use the relations to extend the expressions for the number of faces of neighborly polytopes to the number of cells of levels in neighborly arrangements.

## Introduction

The use of topological methods to study questions in discrete geometry is a well established paradigm, as documented in survey articles (Björner 1995; Živaljević 2017) and books (Matoušek 2008). This paper contributes by viewing questions about splitting finite point sets through the lens of the discrete depth function defined on a corresponding arrangement. To avoid the case analysis needed to distinguish bounded and unbounded cells, we work with arrangements of great-spheres on ${\mathbb {S}}^d$ rather than of hyperplanes in ${\mathbb {R}}^d$. Assuming non-vertical great-spheres (which do not pass through the north-pole and the south-pole) the *depth function* maps every cell of the arrangement to the number of great-spheres that separate the cell from the north-pole.

Aspects of this function have been studied in the past, such as the maximum number of chambers (top-dimensional cells) at a given depth, which relates to counting *k*-*sets* in a set of *n* points, which are subsets of *k* points that can be separated from the remaining $n-k$ points by a straight line; see e.g. Erdős et al. (1973). Giving tight bounds on the number of *k*-sets is still open, with substantial gaps between the current best upper and lower bounds in all dimensions larger than or equal to 2. We propose to focus on the topological aspects of the depth function, in particular the occurrence of critical cells of different types; see Definition 1. In the top dimension, we have a chamber containing the north-pole (a minimum at depth 0), a chamber containing the south-pole (a maximum at depth *n*), and otherwise only non-critical chambers connecting the minimum to the maximum. There is nothing much topological to learn from such a *bi-polar* depth function, but its restrictions to common intersections of great-spheres display a richer topology, which can be studied with methods from discrete Morse theory (Forman 1998) and persistent homology (Edelsbrunner and Harer 2010). The core result in this paper is a system of Dehn–Sommerville type relations for level sets of the depth function. This is different but related to the more direct generalization of the Dehn–Sommerville relations to levels in arrangements proved by Linhart et al. (2000). We refer to Grünbaum (1967, Sect. 9.2) for an introduction to the Dehn–Sommerville relations for convex polytopes. Similar to their classic relatives and the generalization in Linhart et al. (2000), our relations are based on double-counting, but instead counting cells, we take sums of topological indicators. To state the relations, let ${\mathcal {A}}$ be an arrangement of *n* great-spheres in ${\mathbb {S}}^d$, and write ${C}_{k}^{p} ({\mathcal {A}})$ for the number of *p*-cells at depth *k* in ${\mathcal {A}}$. For each *p*-cell, consider the alternating sum of its faces at the same depth, and write $E_{k}^{p} ({\mathcal {A}})$ for the sum of such alternating sums over all *p*-cells at depth *k*. If ${\mathcal {A}}$ is simple (see Sect. 2.1 for a definition), then we have a system of linear relations for $0 \le p \le d$ and $0 \le k \le n-d+p$:

1 $$\begin{aligned} \sum \nolimits _{i=0}^p (-1)^i \genfrac(){0.0pt}1{d-i}{d-p} E_{k}^{i} ({\mathcal {A}})&= {C}_{k}^{p} ({\mathcal {A}}) = \sum \nolimits _{i=0}^p \genfrac(){0.0pt}1{d-i}{d-p} E_{k+i-p}^{i} ({\mathcal {A}}) , \end{aligned}$$

which we refer to as *Dehn–Sommerville–Euler relations*. The system has applications to *cyclic polytopes*—which are convex hulls of finitely many points on the moment curve—and the broader class of *neighborly polytopes*—which are characterized by the property that every $(q-1)$-simplex spanned by $q \le d/2$ vertices is a face of the polytope. A celebrated result in the field is the Upper Bound Theorem proved by McMullen (1970), which states that every cyclic polytope has at least as many faces of any dimension as the convex hull of any other set of *n* points in ${\mathbb {R}}^d$. All cyclic polytopes with *n* vertices in ${\mathbb {R}}^d$ have isomorphic face complexes with a structure that is simple enough to allow for counting the faces, and expressions for these numbers can be found in textbooks, such as Ziegler (1995). In contrast, neighborly polytopes with *n* vertices in ${\mathbb {R}}^d$ can have non-isomorphic face complexes, but they still have the same number of faces in every dimension. Within our framework, the structural simplicity is expressed by having bi-polar restrictions of the depth function to the intersection of any $q \le d/2$ great-spheres. We call an arrangement in ${\mathbb {S}}^d$ that has this property a *neighborly arrangement*. Writing $p = d-q$ and counting only the cells of the subarrangement, ${\mathcal {B}}$, in the intersection of the *q* great-spheres, straightforward topological arguments imply

2 $$\begin{aligned} E_{k}^{p} ({\mathcal {B}})&= \left\{ \begin{array}{ll} 1 &{} \text{ for } k = 0 , \\ 0 &{} \text{ for } 1 \le k \le n+p-d-1 , \\ (-1)^{p} &{} \text{ for } k = n+p-d . \end{array} \right. \end{aligned}$$

Together with the Dehn–Sommerville–Euler relations in (1), this implies expressions in *n*, *d*, *p*, and *k* for the number of *p*-faces, for *every* $0 \le p \le d$, and thus generalizes the result for convex polytopes to levels in neighborly arrangements. Surprisingly, the neighborly property not only determines the number of faces of the convex hull but in fact of every level of the corresponding dual arrangement. The special case of cyclic polytopes, in which the hyperplanes are dual to points on the moment curve, has been solved in Andrzejak and Welzl (2003).

*Outline* Sect. 2 presents the background needed for the results in this paper. Section 3 studies the face and coface structure of a cell in an arrangement. Section 4 uses the technical lemmas in Sect. 3 to prove the system of relations (1), which it compares with the more classic extension of the Dehn–Sommerville relations in Linhart et al. (2000). Section 5 reproduces known bounds on the size of higher-order Voronoi tessellations in two and three dimesions from our system of relations. Section 6 uses (1) to generalize results for neighborly polytopes to neighborly arrangements. Section 7 concludes the paper.

## Background

In this section, we introduce the main geometric and topological concepts studied in this paper: arrangements, depth functions, and sublevel sets.

### Arrangements

**Fig. 1:**
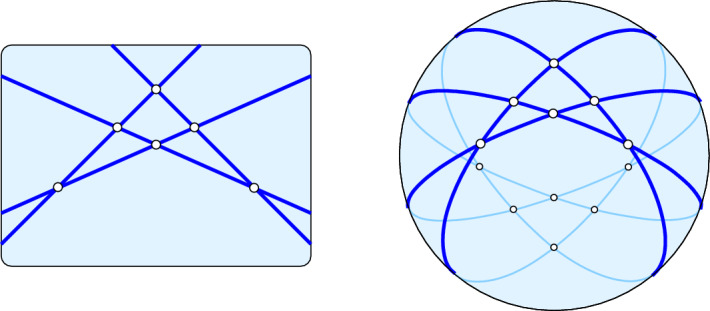
An arrangement of four lines in ${\mathbb {R}}^2$ on the *left* and the corresponding arrangement of four great-circles in ${\mathbb {S}}^2$ on the *right*

As mentioned in Sect. 1, we study the properties of a finite point set in the dual setting, where each point is represented by a non-vertical hyperplane. To further finesse the inconvenience of unbounded cells, we map every point in ${\mathbb {R}}^d$ to a $(d-1)$-dimensional great-sphere and consider the arrangement formed by these great-spheres in ${\mathbb {S}}^d$. Besides having only bounded cells, the great-sphere arrangement is centrally symmetric and thus has two antipodal cells for each bounded cell and each pair of diametrically opposite unbounded cells in the hyperplane arrangement. A possible such transformation maps a point $a = (a_1, a_2, \ldots , a_d) \in {\mathbb {R}}^d$ to the hyperplane defined by the equation $x_d + a_d = a_1 x_1 + a_2 x_2 + \cdots + a_{d-1} x_{d-1}$ and further to the great-sphere in ${\mathbb {S}}^d$ obtained by intersecting the unit-sphere in ${\mathbb {R}}^{d+1}$ with the (*d*-dimensional) hyperplane defined by $x_d + a_d x_{d+1} = a_1 x_1 + a_2 x_2 + \cdots + a_{d-1} x_{d-1}$; see Fig. 1.

Two points in ${\mathbb {S}}^d$ are distinguished: the *north-pole* at the very top and the *south-pole* at the very bottom of the sphere. By construction, none of the great-spheres passes through the two poles. Letting ${\sigma }$ be a great-sphere in ${\mathbb {S}}^d$, we write ${\sigma }^-$ for the closed *lower hemisphere* bounded by $\sigma$, which contains the south-pole, and we write ${\sigma }^+$ for the closed *upper hemisphere*, which contains the north-pole. Letting *A* be the collection of great-spheres, each *cell* in the *arrangement* corresponds to a tri-partition, $A = A^- \sqcup A^0 \sqcup A^+$, such that the cell is the common intersection of the lower hemispheres ${\sigma }^-$, with ${\sigma }\in A^-$, the great-spheres ${\sigma }$, with ${\sigma }\in A^0$, and the upper hemispheres ${\sigma }^+$, with ${\sigma }\in A^+$. We write ${\mathcal {A}}$ for the arrangement defined by *A*, we refer to a cell of dimension *p* as a *p*-*cell*, and for $p = 0$, 1, 2, $d-1$, *d*, we call it a *vertex*, *edge*, *polygon*, *facet*, *chamber*, respectively. The *faces* of a cell are the cells contained in it, which includes the cell itself.

The intersection of great-spheres is again a great-sphere, albeit of a smaller dimension. To avoid any confusion, we will explicitly mention the dimension if it is less than $d-1$. We call the arrangement *simple* if all great-spheres avoid the two poles and the common intersection of any $d-p$ great-spheres is a *p*-dimensional great-sphere in ${\mathbb {S}}^d$. This implies that any *d* great-spheres intersect in a pair of antipodal points, and any $d+1$ or more great-spheres have an empty common intersection. For each $0 \le p \le d$, we write ${C}_{}^{p} = {C}_{}^{p} ({\mathcal {A}})$ for the number of *p*-cells in the arrangement, and ${C}_{}^{p} (n,d)$ for the maximum over all arrangements of *n* great-spheres in ${\mathbb {S}}^d$. Importantly, the number of cells is maximized if the arrangement is simple, and in this case it depends on the number of great-spheres but not on the great-spheres themselves.

#### Proposition 1

(Number of cells) Any simple arrangement of $n \ge d$ great-spheres in ${\mathbb {S}}^d$ has ${C}_{}^{p} (n,d) = 2 \left[ \genfrac(){0.0pt}1{d}{p} \genfrac(){0.0pt}1{n}{d} + \genfrac(){0.0pt}1{d-2}{p-2} \genfrac(){0.0pt}1{n}{d-2} + \cdots + \genfrac(){0.0pt}1{d-2i}{p-2i} \genfrac(){0.0pt}1{n}{d-2i} \right]$ *p*-cells, in which $i = {\left\lfloor p/2 \right\rfloor }$.

The formula for the number of *p*-cells is not new and can be derived from similar formulas for arrangements in *d*-dimensional real projective space (Grünbaum 1967, Sect. 18.1) or in *d*-dimensional Euclidean space (Edelsbrunner 1987, Sect. 1.2).

### Depth function

Given a set *A* of *n* great-spheres in ${\mathbb {S}}^d$, none passing through the two poles, we define the *depth* of a point $x \in {\mathbb {S}}^d$ as the number of great-spheres ${\sigma }\in A$ with $x \in {\sigma }^- \setminus {\sigma }$. In words, the depth of the point is the number of great-spheres that cross the shortest arc connecting *x* to the north-pole. If *x* and *y* are two interior points of the same cell, then they have the same depth. Recalling that ${\mathcal {A}}$ is the arrangement defined by *A*, we introduce the *depth function*, ${{\theta }} :{\mathcal {A}}\rightarrow [0,n]$, which we define by mapping each cell to the depth of its interior points. Depending on the situation, we think of ${{\theta }}$ as a discrete function on the arrangement or a piecewise constant function on ${\mathbb {S}}^d$, namely constant in the interior of every cell in ${\mathcal {A}}$.

Let *c* be a *p*-cell in ${\mathcal {A}}$, with corresponding tri-partition $A^- \sqcup A^0 \sqcup A^+$. The depth of every interior point $x \in c$ is ${{\theta }} (x) = {{\theta }} (c) = {{\#}{A^-}}$, and if the arrangement is simple, then $p = d - {{\#}{A^0}}$. Let $b \subseteq c$ be a face of dimension $i \le p$, with corresponding tri-partition $B^- \sqcup B^0 \sqcup B^+$. We have $B^- \subseteq A^-$, $A^0 \subseteq B^0$, $B^+ \subseteq A^+$, and if the arrangement is simple, we also have $i = d - {{\#}{B^0}}$. Given the depth of *c*, this implies the following bounds on the depth of *b*:

#### Lemma 1

(Depth of face) Let ${\mathcal {A}}$ be a simple arrangement of great-spheres in ${\mathbb {S}}^d$. For every *i*-face, *b*, of a *p*-cell, *c*, we have $\max \{ 0, {{\theta }}(c) + i - p \} \le {{\theta }} (b) \le {{\theta }} (c)$, and both bounds on the depth of *b* are tight.

#### Proof

Since the arrangement is simple, we have ${{\#}{B^-}} \ge {{\#}{A^-}} - [{{\#}{B^0}} - {{\#}{A^0}}] = {{\#}{A^-}} + i - p$, which implies the first inequality. The second inequality follows from ${{\#}{B^-}} \le {{\#}{A^-}}$, which holds for general and not necessarily simple arrangements.

To prove the second inequality is tight, we show the existence of a *p*-cell that shares *b* with *c* and has the same depth as *b*. To this end, consider the tri-partition $(B^+ \cup X) \sqcup (B^0 {\setminus } X) \sqcup B^-$, in which $X \subseteq B^0$ has cardinality $p-i$. The cell defined by this tri-partition is non-empty because it contains *b* as a face. Furthermore, this cell has dimension *p* and the same depth as *b*. The proof that the first inequality is tight is symmetric and omitted. $\square$

To relate this concept to the prior literature, we mention that Edelsbrunner (1987, Chapter 3) introduces the *k*-*th level* of an arrangement of *n* non-vertical hyperplanes in *d* dimensions as the points $x \in {\mathbb {R}}^d$ below fewer than *k* and above fewer than $n-k$ of the hyperplanes. In other words, the *k*-th level consists of all facets at depth $k-1$ and all their faces. Assuming the arrangement is simple, Lemma 1 implies that a *p*-cell belongs to the *k*-th level iff its depth is between $k-d+p$ and $k-1$.

### Sublevel sets

Assume that ${\mathcal {A}}$ has at least one vertex, which in the simple case is implied by $n \ge d$. For $0 \le k \le n$, we write ${\mathcal {A}}_k = {{\theta }}^{-1} [0,k]$ for the *sublevel set* of ${{\theta }}$ at *k*. It consists of all cells in ${\mathcal {A}}$ whose depth is *k* or less. Recall that ${{\theta }}$ is *monotonic*, by which we mean that the depth of every cell is at least as large as the depth of any of its faces. It follows that ${\mathcal {A}}_k$ is a complex, with well defined *Euler characteristic*:

3 $$\begin{aligned} {\chi }{({{\mathcal {A}}_k})}&= \sum \nolimits _{c \in {\mathcal {A}}_k} (-1)^{\mathrm{dim\,}{c}} . \end{aligned}$$

The right-hand side of (3) explains how the Euler characteristic changes from ${\mathcal {A}}_{k-1}$ to ${\mathcal {A}}_k$, namely by adding the alternating sum of all cells at depth *k*. By Lemma 1, every cell at depth *k* is a face of a chamber at depth *k*. We can therefore construct ${\mathcal {A}}_k$ from ${\mathcal {A}}_{k-1}$ by adding all chambers at depth *k* together with their faces at the same depth. This motivates the following two definitions.

#### Definition 1

(*Relative Euler and depth characteristic*) For a cell $c \in {\mathcal {A}}$, let $F = F(c)$ be the complex of faces, which includes *c*, and let $F_0 \subseteq F$ be a subcomplex. The *relative Euler characteristic* of the pair of complexes is ${\chi }{({F,F_0})} = \sum \nolimits _{b \in F {\setminus } F_0} (-1)^{\mathrm{dim\,}{b}}$.

If $F_0$ is the set of faces $b \subseteq c$ with ${{\theta }} (b) < {{\theta }} (c)$, denoted $U = U (c)$, we call ${\varepsilon }{({c})} = {\chi }{({F,U})}$ the *depth characteristic* of *c*, and we call *c* *critical* for ${{\theta }}$ if ${\varepsilon }{({c})} \ne 0$.

For example, if all faces have the same depth as *c*, then the depth characteristic of *c* is ${\varepsilon }{({c})} = {\chi }{({F,\emptyset })} = 1$, and if all proper faces have depth strictly less than *c*, then the depth characteristic of *c* is ${\varepsilon }{({c})} = {\chi }{({F,F {\setminus } \{c\}})} = (-1)^{\mathrm{dim\,}{c}}$. In both cases, *c* is critical.

#### Lemma 2

(Relative and absolute Euler characteristic) Let $F = F(c)$ be the face complex of a cell, *c*, in an arrangement, and let $F_0 \subseteq F$ be a subcomplex. Then the relative Euler characteristic of the pair is ${\chi }{({F,F_0})} = 1 - {\chi }{({F_0})}$.

#### Proof

By definition, ${\chi }{({F,F_0})} + {\chi }{({F_0})}$ is the sum of $(-1)^{\mathrm{dim\,}{b}}$ over all cells $b \in F \setminus F_0$ as well as all $b \in F_0$, and therefore over all $b \in F$. Hence, this sum is ${\chi }{({F})}$, which is equal to 1 because *c* is closed and convex. The claimed equation follows. $\square$

We write ${C}_{k}^{p} = {C}_{k}^{p} ({\mathcal {A}})$ for the number of *p*-cells at depth *k*, and $E_{k}^{p} = E_{k}^{p} ({\mathcal {A}}) = \sum _c {\varepsilon }{({c})}$ for the sum of depth characteristics over all *p*-cells at depth *k*. To see the motivation behind taking sums of depth characteristics, consider the subcomplex of cells at depth at most *k* in a *p*-dimensional subarrangement of the *d*-dimensional arrangement. It is pure *p*-dimensional, by which we mean that every cell in this subcomplex is a face of a *p*-cell. Furthermore, the Euler characteristic of this pure complex is the sum of depth characteristics of its *p*-cells. In other words, we can construct the subarrangement by adding its *p*-cells in the order of non-decreasing depth. Whenever we add a *p*-cell, *c*, we also add the yet missing faces, and we know that ${\varepsilon }{({c})}$ is the increment to the Euler characteristic of the subcomplex. Hence, $E_{k}^{p}$ is the increment to the total Euler characteristic of the subcomplexes in the *p*-dimensional subarrangements when we add the *p*-cells at depth *k* together with their yet missing faces.

## Local configurations

Most arguments in the subsequent technical sections accumulate local quantities, each counting faces or cofaces of a cell. In a simple arrangement, the coface structure depends only on the dimension, so we study it first.

### Coface structure

In the generic case, the local neighborhood of a vertex in an arrangement in ${\mathbb {S}}^{d}$ looks like that of the origin in the arrangement of the *d* coordinate planes in ${\mathbb {R}}^{d}$. Each of these $(d-1)$-planes bounds an open half-space in which the corresponding coordinate is strictly negative. Accordingly, we define the *depth* of a point $x \in {\mathbb {R}}^{d}$ as the number of negative coordinates, and the *depth* of a cell in the arrangement as the depth of its interior points. To study this arrangement, consider $[-1, 1]^{d} \subseteq {\mathbb {R}}^{d}$ and let ${S}_{}^{p}{({d})}$ be the number of *q*-sides of the *d*-cube, in which we write $q = d-p$. The dual correspondence provides an incidence reversing bijection between the *p*-cells of the arrangement and the *q*-sides of the cube.

**Fig. 2:**
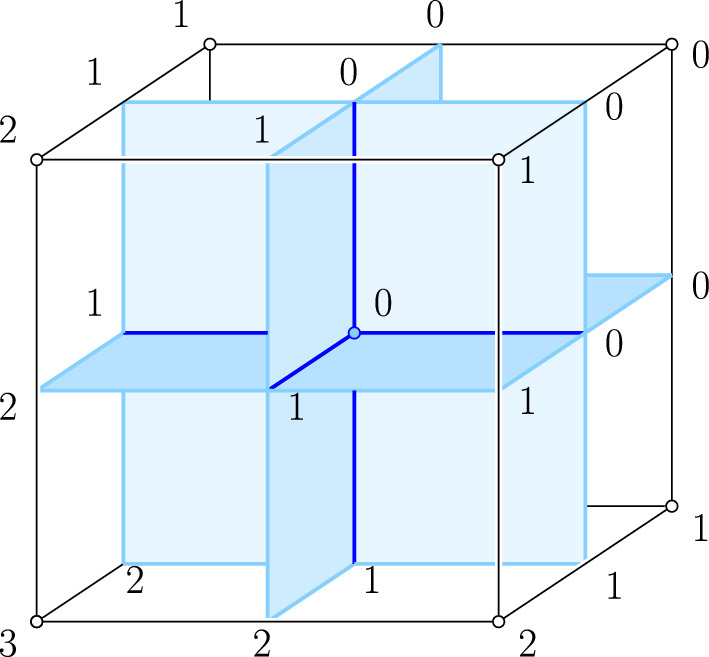
The neighborhood of the origin in ${\mathbb {R}}^3$ and the dual cube centered at the origin. The labels of the sides are the depths of the corresponding cells in the arrangement of coordinate planes

We label each side with the depth of the corresponding cell in the arrangement, and write ${S}_{k}^{p}{({d})}$ for the number of *q*-sides labeled *k*. As illustrated in Fig. 2, this amounts to labeling $${S}_{k}^{d}{({d})} = \left( {\begin{array}{c}d\\ k\end{array}}\right) $$ vertices with *k*, for $0 \le k \le d$, and labeling each side with the minimum label of its vertices. Note that the label of a *q*-side cannot exceed $d - q = p$.

#### Lemma 3

(Coface structure of vertex) Consider the arrangement defined by the *d* coordinate planes in ${\mathbb {R}}^d$.

(i) For $0 \le k \le p \le d$, the number of *p*-cells at depth *k* is ${S}_{k}^{p}{({d})} = \genfrac(){0.0pt}1{d-k}{d-p} \genfrac(){0.0pt}1{d}{k}$.
(ii) There is one cell at depth *d*, namely the negative orthant, and for $0 \le k < d$, the alternating sum of cells at depth *k* vanishes; that is: $\sum \nolimits _{p=k}^d (-1)^p {S}_{k}^{p}{({d})} = 0$.

#### Proof

The *p*-cells counted in (i) correspond to the *q*-sides with label *k*, in which $p+q = d$. To count these *q*-sides, we recall that the *d*-cube has $$\left( {\begin{array}{c}d\\ k\end{array}}\right) $$ vertices at depth *k*. For each such vertex, *u*, consider the largest side for which *u* is the vertex with minimum label. This largest side is a cube of dimension $d-k$, which contains $$\left( {\begin{array}{c}d-k\\ q\end{array}}\right) $$ *q*-sides incident to *u*. We thus get

4 $$\begin{aligned} {S}_{k}^{p}{({d})}&= \genfrac(){0.0pt}1{d-k}{q} \genfrac(){0.0pt}1{d}{k} = \genfrac(){0.0pt}1{d-k}{d-p} \genfrac(){0.0pt}1{d}{k} \end{aligned}$$

*q*-sides with label *k*, which proves (i).

To see (ii), consider a $(d-k)$-cube with label *k*. The alternating sum of sides with the same label is $$\sum \nolimits _{q=0}^{d-k} (-1)^q \left( {\begin{array}{c}d-k\\ q\end{array}}\right) $$, which vanishes for $d-k > 0$, and equals 1 for $d-k = 0$. Likewise, the sum of alternating sums over all $(d-k)$-sides with label *k* vanishes for $d-k > 0$ and equals 1 for $k = d$. This implies (ii) by duality. $\square$

It is easy to generalize Lemma 3 from a vertex to a cell of dimension $i \ge 0$. To see this geometrically, we slice the *i*-cell and its cofaces with a $(d-i)$-plane orthogonal to the *i*-cell. In this slice, the *i*-cell appears as a vertex, and each coface of dimension *p* appears as a $(p-i)$-cell.

#### Corollary 1

(Coface structure of cell) Consider the arrangement defined by the *d* coordinate planes in ${\mathbb {R}}^d$, and let *c* be an *i*-cell at depth $0 \le \ell \le i$.

(i) For $0 \le k-\ell \le p-i \le d-i$, the number of *p*-cells at depth *k* that contain *c* is ${S}_{k-\ell }^{p-i}{({d-i})} = \genfrac(){0.0pt}1{d-i-k+\ell }{d-p} \genfrac(){0.0pt}1{d-i}{k-\ell }$.
(ii) There is one cell at depth *d*, and for $\ell \le k < d$, the alternating sum of cells at depth *k* that contain *c* vanishes; that is: $\sum \nolimits _{p=k}^d (-1)^p {S}_{k-\ell }^{p-i}{({d-i})} = 0$.

### Face structure

The face structure of a cell in a simple arrangement is not quite as predictable as its coface structure. Nevertheless, we can say something about it. As before, we write $F = F(c)$ for the face complex of a cell, *c*, and we let $F_0 \subseteq F$ be a subcomplex. Furthermore, we write

5 $$\begin{aligned} {\textrm{X}{({F,F_0})}}&= \sum \nolimits _{b \in F \setminus F_0} (-1)^{\mathrm{dim\,}{b}} {\chi }{({F(b),F_0 \cap F(b)})} \end{aligned}$$

for the alternating sum of relative Euler characteristics.

#### Lemma 4

(Face structure of cell) Let *c* be a cell in a simple arrangement of great-spheres in ${\mathbb {S}}^d$, and let $F_0 \subseteq F(c)$ be a subcomplex of the face complex of the cell. Then ${\textrm{X}{({F,F_0})}} = 1$ if $F_0 \ne F$ and ${\textrm{X}{({F,F_0})}} = 0$ if $F_0 = F$.

#### Proof

If $F_0 = F$, then ${\textrm{X}{({F,F_0})}}$ is a sum without terms, which is 0. We can therefore assume $F_0 \ne F$, which implies $c \in F {\setminus } F_0$. Fix a cell $a \in F {\setminus } F_0$ with dimension $i = \mathrm{dim\,}{a}$ less than or equal to $p = \mathrm{dim\,}{c}$. It contributes $(-1)^{i+j}$ for every *j*-cell $b \in F \setminus F_0$ that contains *a* as a face. The contribution of *a* to ${\textrm{X}{({F,F_0})}}$ is therefore $$(-1)^i \sum \nolimits _{j=1}^p (-1)^j \left( {\begin{array}{c}p-i\\ j-i\end{array}}\right) $$, which vanishes for all $i < p$ and is equal to 1 for $i=p$. Hence, the only non-zero contribution to ${\textrm{X}{({F,F_0})}}$ is for $a = c$, which implies the claim. $\square$

There is a symmetric form of the lemma, which we get by introducing the *codepth function*, ${{\vartheta }} :{\mathcal {A}}\rightarrow [0,n]$ defined by ${{\vartheta }} (x) = n - q - {{\theta }} (x)$, where *q* is the number of great-spheres that pass through *x*. Observe that ${{\vartheta }} (x)$ is the number of great-spheres that cross the shortest arc connecting *x* to the south-pole. We write ${B}_{\ell }^{p} ({\mathcal {A}})$ for the number of *p*-cells with codepth $\ell$. If the arrangement is simple, then

6 $$\begin{aligned} {B}_{\ell }^{p} ({\mathcal {A}})&= {C}_{k}^{p} ({\mathcal {A}}) , \text{ with } k + \ell + (d-p) = n . \end{aligned}$$

Indeed, there are $d-p$ great-spheres that contain a *p*-cell, *c*, and if *k* great-spheres pass above *c*, then $\ell = n - (k+d-p)$ great-spheres pass below *c*. Recall that ${\varepsilon }{({c})} = {\chi }{({F,U})}$ is the depth characteristic, in which $F = F(c)$ is the face complex, and $U \subseteq F$ is the subcomplex of faces at depth strictly less than ${{\theta }} (c)$. Symmetrically, we call ${\delta }{({c})} = {\chi }{({F,L})}$ the *codepth characteristic* of *c*, in which $F = F(c)$ as before, and $L \subseteq F$ is the subcomplex of faces at codepth strictly less than ${{\vartheta }} (c)$. In a simple arrangement, the two characteristics agree on even-dimensional cells, and they are the negative of each other for odd-dimensional cells.

#### Lemma 5

(Depth and codepth characteristics) For a *p*-cell in a simple arrangement of great-spheres, we have ${\delta }{({c})} = (-1)^p {\varepsilon }{({c})}$.

#### Proof

The boundary of *c* is a $(p-1)$-sphere, which is decomposed by the complex of proper faces of *c*. We write *L* for the proper faces with codepth strictly less than ${{\vartheta }} (c)$, and *U* for the proper faces with depth strictly less than ${{\theta }} (c)$. *L* and *U* exhaust the proper faces of *c*. More precisely, *L* and *U* partition the $(p-1)$-faces, and each of the two subcomplexes is the closure of its set of $(p-1)$-faces. Hence, $L \cap U$ consists of all $(p-2)$-faces shared by a $(p-1)$-face in *L* and another $(p-1)$-face in *U*, together with all faces of these $(p-2)$-faces. Since the arrangement is simple, the cells in $L \cap U$ decompose a $(p-2)$-manifold.

- Case 1: *p* is odd. Then $L \cap U$ decomposes an odd-dimensional manifold. By Poincaré duality, ${\chi }{({L \cap U})} = 0$. The Euler characteristic of the boundary of *c* is 2, which implies ${\chi }{({L})} + {\chi }{({U})} - {\chi }{({L \cap U})} = {\chi }{({L})} + {\chi }{({U})} = 2$. By Lemma 2, ${\varepsilon }{({c})} = 1 - {\chi }{({U})}$ and therefore ${\delta }{({c})} = 1 - {\chi }{({L})} = 1 - [2-{\chi }{({U})}] = -{\varepsilon }{({c})}$, as claimed.
- Case 2: *p* is even. The boundary of *c* is an odd-dimensional sphere, so its Euler characteristic vanishes. By Alexander duality, ${\chi }{({L})} = {\chi }{({U})}$, and by Lemma 2, ${\varepsilon }{({c})} = 1 - {\chi }{({U})}$ and ${\delta }{({c})} = 1 - {\chi }{({L})}$, which implies ${\delta }{({c})} = {\varepsilon }{({c})}$, as claimed.$\square$

## Relations

In this section, we prove linear relations for the cells at given depths. The relations are similar to the classic Dehn–Sommerville relations for convex polytopes, and we prove them the same way by straightforward double counting; see Grünbaum (1967, Sect. 9.2). We begin with the easy bi-polar case.

### Bi-polar depth functions

We recall that the depth function on an arrangement of great-spheres is bi-polar if there is a chamber above all great-spheres. By construction, the arrangement and its depth function are antipodal, which implies that there is also a chamber below all great-spheres. With the great-spheres given in ${\mathbb {S}}^d$, the depth function on ${\mathbb {S}}^d$ is necessarily bi-polar, but its restrictions to subarrangements inside the common intersection of one or more great-spheres are not necessarily bi-polar.

#### Theorem 1

(Bi-polar depth functions) Let ${\mathcal {A}}$ be a simple arrangement of $n \ge d$ great-spheres in ${\mathbb {S}}^d$, let ${\mathcal {B}}$ be the *p*-dimensional subarrangement inside the intersection of $d-p$ of the great-spheres, and assume that the restriction of the depth function to ${\mathcal {B}}$ is bi-polar. Then

7 $$\begin{aligned} E_{k}^{p} ({\mathcal {B}})&= \left\{ \begin{array}{ll} 1 &{} \text{ for } k = 0 , \\ 0 &{} \text{ for } 1 \le k \le n-d+p-1 , \\ (-1)^p &{} \text{ for } k = n-d+p . \end{array} \right. \end{aligned}$$

#### Proof

Let $c_N$ be the (*p*-dimensional) chamber at depth 0 in ${\mathcal {B}}$, and let $c_S$ be the antipodal chamber at depth $n-d+p$. We write ${\mathbb {S}}^p$ for the intersection of the $d-p$ great-spheres, fix a point $N \in {\mathbb {S}}^p$ inside the interior of $c_N$, and let $S \in {\mathbb {S}}^p$ in the interior of $c_S$ be the antipodal point. We partition ${\mathbb {S}}^p \setminus \{N,S\}$ into open fibers, each half a great-circle connecting *N* to *S*. Along each fiber, the depth is non-decreasing. Consider the set of fibers that intersect a chamber $c \ne c_N, c_S$. They partition the boundary of *c* into the *upper boundary*, along which the fibers enter the chamber, the *lower boundary*, along which the fibers exit the chamber, and the *silhouette*, along which the fibers touch but do not enter the chamber. Since *c* is *p*-dimensional and spherically convex (the common intersection of closed hemispheres) this implies that the silhouette is a $(p-2)$-sphere, and the upper and lower boundaries are open $(p-1)$-balls. The depth characteristic of *c* is $(-1)^{p-1}$—for the open lower boundary—plus $(-1)^p$—for the chamber itself. It follows that the depth characteristic of *c* vanishes, and so does the depth characteristic of every other chamber, except for $c_N$ and $c_S$. Because $c_N$ has the same depth as its entire boundary, we have ${\varepsilon }{({c_N})} = 1$, and because $c_S$ has larger depth than its entire boundary, we have ${\varepsilon }{({c_S})} = (-1)^p$. This implies (7). $\square$

### Alternating sums of depth characteristics

In the general case, the restrictions of the depth function to subarrangements are not necessarily bi-polar. The depth characteristics may therefore violate (7), but they satisfy a system of linear relations, as we prove next.

#### Theorem 2

(Dehn–Sommerville–Euler for levels) Let ${\mathcal {A}}$ be a simple arrangement of $n \ge d$ great-spheres in ${\mathbb {S}}^d$. Then for every dimension $0 \le p \le d$, we have

8 $$\begin{aligned} \sum \nolimits _{i=0}^p (-1)^i \genfrac(){0.0pt}1{d-i}{p-i} E_{k}^{i} ({\mathcal {A}})&= {C}_{k}^{p} ({\mathcal {A}})\nonumber \\&= \sum \nolimits _{i=0}^p \genfrac(){0.0pt}1{d-i}{p-i} E_{k+i-p}^{i} ({\mathcal {A}}) \quad \text{ for }\quad 0 \le k \le n-d+p. \end{aligned}$$

#### Proof

Let *c* be a *p*-cell at depth *k*, let $F = F(c)$ be the face complex of *c*, and let $U \subseteq F$ be the subcomplex of faces at depth strictly less than *k*. Note that *U* does not contain *c*, so $U \ne F$, and Lemma 4 implies ${\textrm{X}{({F,U})}} = 1$. Taking the sum over all *p*-cells at depth *k* thus gives the number of such *p*-cells, which is ${C}_{k}^{p} ({\mathcal {A}})$. By Corollary 1 (i), a single *i*-cell contributes to the alternating sums of ${S}_{0}^{p-i}{({d-i})} = \genfrac(){0.0pt}1{d-i}{p-i}$ *p*-cells, which implies that the first sum in (8) is the total alternating sum of depth characteristics over all cells at depth *k* and dimension at most *p*.

The second relation in (8) is the upside-down version of the first relation. Indeed, we can substitute codepth for depth and get the following relation using the notation of Sect. 3.2:

9 $$\begin{aligned} {B}_{\ell }^{p} ({\mathcal {A}})&= \sum \nolimits _{i=0}^p (-1)^i \genfrac(){0.0pt}1{d-i}{p-i} {D}_{\ell }^{i} ({\mathcal {A}}) . \end{aligned}$$

To translate this back in term of depth, we set $\ell = n - (k+d-p)$ so that a *p*-cell at codepth $\ell$ has depth $n - (\ell +d-p) = k$. Hence, ${B}_{\ell }^{p} ({\mathcal {A}}) = {C}_{k}^{p} ({\mathcal {A}})$. To write the *D*s in terms of the *E*s, we multiply with $(-1)^i$ because of Lemma 5, and we change the index from $\ell = n - (k+d-p)$ to $k+i-p = n - (\ell +d-i)$ because of (6). This gives the right relation in (8). $\square$

As an example consider the case $d=2$. We get equations (10)–(12) by setting $p = 0,1,2$ in (8):

10 $$\begin{aligned}&E_{k}^{0} = {C}_{k}^{0} = E_{k}^{0} , \end{aligned}$$

11 $$\begin{aligned}&2 E_{k}^{0} - E_{k}^{1} = {C}_{k}^{1} = 2 E_{k-1}^{0} + E_{k}^{1} , \end{aligned}$$

12 $$\begin{aligned}&E_{k}^{0} - E_{k}^{1} + E_{k}^{2} = {C}_{k}^{2} = E_{k-2}^{0} + E_{k-1}^{1} + E_{k}^{2} , \end{aligned}$$

Equation (10) just says that the depth characteristic of every vertex is 1. (11) implies $E_{k}^{1} = E_{k}^{0} - E_{k-1}^{0}$, and (12) implies $E_{k}^{1} + E_{k-1}^{1} = E_{k}^{0} - E_{k-2}^{0}$, which follows from the relation implied by (11). Note that adding the depth characteristics of the edges gives a telescoping series, which implies $E_{0}^{1} + E_{1}^{1} + \ldots + E_{k}^{1} = E_{k}^{0}$.

### Alternating sums of cells

For comparison, we state the more traditional version of the Dehn–Sommerville relations, which apply to cell complexes; see Mulmuley (1993) and Linhart et al. (2000, Theorem 1). It counts the *p*-cells at depth *k*, which together with all their faces form a cell complex. For each dimension $0 \le i \le p$, this includes all *i*-cells at depths $k+i-p$ to *k*.

#### Proposition 2

(Dehn–Sommerville for levels) Let ${\mathcal {A}}$ be a simple arrangement of $n \ge d$ great-spheres in ${\mathbb {S}}^d$. For every dimension $0 \le p \le d$, we have

13 $$\begin{aligned} {C}_{k}^{p} ({\mathcal {A}})&= \sum \nolimits _{i=0}^p (-1)^i \genfrac(){0.0pt}1{d-i}{d-p} \sum \nolimits _{j=0}^{p-i} \genfrac(){0.0pt}1{p-i}{p-i-j} {C}_{k+i-p+j}^{i} ({\mathcal {A}}) \quad \mathrm{for~} 0 \le k \le n-d+p. \end{aligned}$$

We get a non-trivial relation in (13) for $p = 1$, which asserts ${C}_{k}^{1} = d {C}_{k-1}^{0} + d {C}_{k}^{0} - {C}_{k}^{1}$. Indeed, twice the number of edges is the sum of vertex degrees. For $p=2$, we get

14 $$\begin{aligned} {C}_{k}^{2}&= \genfrac(){0.0pt}1{d}{2} {C}_{k}^{0} - (d-1) {C}_{k}^{1} + {C}_{k}^{2} + (d-1)d {C}_{k-1}^{0} - (d-1) {C}_{k-1}^{1} + \genfrac(){0.0pt}1{d}{2} {C}_{k-2}^{0} , \end{aligned}$$

in which the polygons cancel and the rest is equivalent to the relation for $p = 1$. More generally, the term on left-hand side of (13) cancels whenever *p* is even.

## Application to higher-order Voronoi tessellations

In this section, we give evidence for the unifying power of the system of Dehn–Sommerville–Euler relations by rederiving cell-counting formulas for higher-order Voronoi tessellations proved in Biswas et al. (2021) and Lee (1982). The difference forms of the relations are particularly convenient, which we present in dimensions 3 and 4.

### Two dimensions

Before discussing the 2-dimensional order-*k* Voronoi tessellations, we introduce the 3-dimensional difference relations implied by Theorems 1 and 2.

#### Corollary 2

(Difference relations in ${\mathbb {S}}^3$) Let ${\mathcal {A}}$ be a simple arrangement of $n \ge 3$ great-spheres in ${\mathbb {S}}^3$. Then

15 $$\begin{aligned} E_{k}^{1} ({\mathcal {A}})&= \tfrac{3}{2} [ E_{k}^{0} ({\mathcal {A}}) - E_{k-1}^{0} ({\mathcal {A}})], \quad \mathrm{~~for~} 0 \le k \le n, \end{aligned}$$

16 $$\begin{aligned} E_{k}^{2} ({\mathcal {A}})&= \tfrac{1}{3} [E_{k}^{1} ({\mathcal {A}}) - E_{k-1}^{1} ({\mathcal {A}})] + 2 , \quad \mathrm{~~for~} 0 \le k \le n, \end{aligned}$$

17 $$\begin{aligned} E_{k}^{3} ({\mathcal {A}})&= \left\{ \begin{array}{ll} 1 &{} \textrm{for }\, k = 0, \\ 0 &{} \textrm{for} \,1 \le k \le n-1, \\ -1 &{} \textrm{for}\, k = n. \end{array} \right. \end{aligned}$$

#### Proof

We get (15) by setting $d=3$ and $p=1$ in (8) and (17) by setting $d=p=3$ in (7). To get (16), we begin by setting $d=p=3$ in (8), which gives

18 $$\begin{aligned} E_{k}^{2} - E_{k-1}^{2}&= [E_{k-3}^{0} -E_{k}^{0}] + [E_{k-2}^{1}+E_{k}^{1}] + 2 E_{k}^{3}\nonumber \\&= \tfrac{1}{3} [E_{k}^{1}-2E_{k-1}^{1} +E_{k-2}^{1}] + 2E_{k}^{3} , \end{aligned}$$

in which we use $E_{k-3}^{0}-E_{k}^{0} = -\tfrac{2}{3} [E_{k}^{1}+E_{k-1}^{1}+E_{k-2}^{1}]$ implied by (15). Moving $E_{k-1}^{2}$ to the right-hand side and substituting it recursively implies (16) because $\sum _{\ell =0}^k E_{\ell }^{3} = 1$ by (17). $\square$

If every 2-dimensional subarrangement is bipolar, then each arrangement of $n-1$ great-circles inside a great-sphere has polygons of predictable depth characteristics, namely a minimum (with depth characteristic 1) at depth 0, a maximum (with depth characteristic 1) at depth $n-1$, and otherwise only non-critical polygons connecting the minimum to the maximum. Hence,

19 $$\begin{aligned} E_{k}^{2} ({\mathcal {A}})&= \left\{ \begin{array}{ll} n &{} \hbox { for } k=0, n-1, \\ 0 &{} \hbox { for } 1 \le k \le n-2, \end{array} \right. \end{aligned}$$

20 $$\begin{aligned} E_{k}^{1} ({\mathcal {A}})&= 3n - 6(k+1) \text{ for } 0 \le k \le n-2, \end{aligned}$$

21 $$\begin{aligned} E_{k}^{0} ({\mathcal {A}})&= 2n(k+1) - 4 \genfrac(){0.0pt}1{k+2}{2} \text{ for } 0 \le k \le n-3 , \end{aligned}$$

in which we get (20) from (19) and (16), and we get (21) from (20) and (15). For values of *k* outside the given limits, the sums of Euler characteristics are zero.

As defined in Shamos and Hoey (1975), the *order-k* *Voronoi tessellation* of *n* points in ${\mathbb {R}}^2$ is a decomposition of the plane into closed convex regions such that any two points in a region share the same *k* nearest points in the given set; but see also Toth (1976). It can be obtained by mapping each of the *n* points, $u = (u_1, u_2)$, to the plane $x_3 = u_1 x_1 + u_2 x_2 + \tfrac{1}{2} (u_1^2 + u_2^2)$, forming the arrangement of the *n* planes, and projecting the chambers at depth *k* to the regions of the tessellation. The boundaries of the regions are obtained by projecting the edges at depth $k-1$ and the vertices at depths $k-2$ and $k-1$. Lee counted the regions, edges, and vertices in these tessellations (Lee 1982), and found that the numbers depend on *n* and *k* but barely on how the points are placed in the plane. Indeed, if we modify the setting slightly by turning the planes into great-spheres—as explained in Sect. 2—then a general position assumption suffices for these numbers to depend solely on *n* and *k*. Using Theorem 2 and the expressions for $E_{\ell }^{p}$ in the case of bipolar 2-dimensional subarrangements given in (21), (20), (19), (17), we get

22 $$\begin{aligned}&{C}_{k-2}^{0} + {C}_{k-1}^{0} = E_{k-2}^{0} + E_{k-1}^{0} = 2(n-k) (2k-1) - 2k , \end{aligned}$$

23 $$\begin{aligned}&{C}_{k-1}^{1} = 3 E_{k-2}^{0} + E_{k}^{1} = 3(n-k) (2k - 1) - 3k, \end{aligned}$$

24 $$\begin{aligned}&{C}_{k}^{3} = E_{k-3}^{0} + E_{k-2}^{0} + E_{k-1}^{0} + E_{k}^{0} = (n-k)(2k-1) - k+2 \end{aligned}$$

for the number of vertices, edges, and regions. Modulo the difference between ${\mathbb {R}}^2$ and ${\mathbb {S}}^2$, these are the same expressions as in Lee (1982).

### Three dimensions

Before discussing the 3-dimensional order-*k* Voronoi tessellations, we introduce the 4-dimensional difference relations implied by Theorems 1 and 2.

#### Corollary 3

(Difference relations in ${\mathbb {S}}^4$) Let ${\mathcal {A}}$ be a simple arrangement of $n \ge 4$ great-spheres in ${\mathbb {S}}^4$. Then

25 $$\begin{aligned} E_{k}^{1} ({\mathcal {A}})&= 2 [ E_{k}^{0} ({\mathcal {A}}) - E_{k-1}^{0} ({\mathcal {A}})], \text{ for } 0 \le k \le n, \end{aligned}$$

26 $$\begin{aligned} E_{k}^{2} ({\mathcal {A}})&= \tfrac{1}{2} [E_{k}^{1} ({\mathcal {A}}) - E_{k-1}^{1} ({\mathcal {A}})] + \sum \nolimits _{\ell =0}^k E_{\ell }^{3}, \text{ for } 0 \le k \le n, \end{aligned}$$

27 $$\begin{aligned} E_{k}^{4} ({\mathcal {A}})&= \left\{ \begin{array}{ll} 1 &{} \text{ for } k = 0, n, \\ 0 &{} \text{ for } 1 \le k \le n-1. \end{array} \right. \end{aligned}$$

#### Proof

We get (25) by setting $d=4$ and $p=1$ in (8), and we get (27) by setting $d=p=4$ in (7). To get (26), we begin by setting $d=4$ and $p=3$ in (8), which gives

28 $$\begin{aligned} E_{k}^{2} - E_{k-1}^{2}&= 2[E_{k-3}^{0}-E_{k}^{0}] + \tfrac{3}{2}[E_{k-2}^{1}+E_{k}^{1}] = \tfrac{1}{2} [E_{k}^{1}-2E_{k-1}^{1}+E_{k-2}^{1}] + E_{k}^{3} , \end{aligned}$$

in which we use $2 [E_{k-3}^{0} - E_{k}^{0}] = - [E_{k}^{1}+E_{k-1}^{1}+E_{k-2}^{1}]$ implied by (25). Moving $E_{k-1}^{2}$ to the right-hand side and substituting iteratively, we get (26). $\square$

Note the absence of any relation for $E_{k}^{3}$. However, if we assume that all 3-dimensional subarrangements are bipolar, there is additional information about the facets and therefore also about the polygons:

29 $$\begin{aligned} E_{k}^{3} ({\mathcal {A}})&= \left\{ \begin{array}{rl} n &{} \hbox { for } k = 0, \\ 0 &{} \hbox { for } 1 \le k \le n-2, \\ -n &{} \hbox { for } k = n-1, \end{array} \right. \end{aligned}$$

30 $$\begin{aligned} E_{k}^{2} ({\mathcal {A}})&= \tfrac{1}{2} [ E_{k}^{1} ({\mathcal {A}}) - E_{k-1}^{1} ({\mathcal {A}})] + n, \hbox { for } 0 \le k \le n-2 , \end{aligned}$$

in which we get (30) from (29) and (26).

By straightforward generalization from 2 to 3 dimensions, the *order-k Voronoi tessellation* of *n* points in ${\mathbb {R}}^3$ decomposes space into convex regions, each associated with the *k* nearest of the *n* points. In analogy to the 2-dimensional case, we map the points to 3-planes in ${\mathbb {R}}^4$—or to great-spheres in ${\mathbb {S}}^4$—so that the tessellation is the projection of a subset of the cells. Despite this similarity, the expressions for the number of cells of the 2-dimensional tessellations derived by Lee (1982) have been extended to 3 dimensions only recently. The main reason for such delay is that the number of cells do not only depend on *n* and *k*, but also on how the points are distributed in space. Indeed, compared to the 2-dimensional case, we have the same number of relations but one more variable. Specifically, we have relations (25), (30), (29), (27), and we count vertices, edges, polygons, and (3-dimensional) regions, which are obtained by projecting the ${C}_{k-1}^{0} + {C}_{k-2}^{0} + {C}_{k-3}^{0}$ vertices at depths $k-1, k-2, k-3$, the ${C}_{k-1}^{1} + {C}_{k-2}^{1}$ edges at depths $k-1, k-2$, the ${C}_{k-1}^{2}$ polygons at depth $k-1$, and the ${C}_{k}^{4}$ chambers at depth *k*. Using Theorem 2 and the four mentioned relations for bipolar 3-dimensional subarrangements, we get

31 $$\begin{aligned}&E_{k-3}^{0} + E_{k-2}^{0} + E_{k-1}^{0} = \textbf{E}_{k-3}^{2} + \textbf{E}_{k-2}^{2} + \textbf{E}_{k-1}^{2} - \tfrac{n}{2} [3k^2 - 3k + 2], \end{aligned}$$

32 $$\begin{aligned}&4 E_{k-2}^{0} - E_{k-2}^{1} + 4E_{k-1}^{0} - E_{k-1}^{1} = 2 \textbf{E}_{k-2}^{2} + 4 \textbf{E}_{k-1}^{2} + 2 \textbf{E}_{k}^{2} - 2n [2k^2-2k+1], \end{aligned}$$

33 $$\begin{aligned}&6E_{k-1}^{0} - 3E_{k-1}^{1} + E_{k-1}^{2} = \textbf{E}_{k-2}^{2} + E_{k-1}^{2} - 3n [k^2-k] , \end{aligned}$$

34 $$\begin{aligned}&E_{k}^{0} - E_{k}^{1} + E_{k}^{2} - E_{k}^{3} + E_{k}^{4} = \textbf{E}_{k-2}^{2} - \tfrac{n}{2} [k^2 - k + 2] \end{aligned}$$

for the number of vertices, edges, polygons, and regions in the order-*k* Voronoi tessellation for $1 \le k \le n-1$, in which $\textbf{E}_{k}^{2} = \sum \nolimits _{m=0}^k \sum \nolimits _{\ell =0}^m E_{\ell }^{2}$. To see that these are the same expressions as in Biswas et al. (2021), we note that $\textbf{E}_{k}^{2} = N_{k+1}$ and $E_{k}^{2} = J_{k+1}$ in the notation of that paper.

## Application to neighborly arrangements

Recall that an arrangement in ${\mathbb {S}}^d$ is neighborly if the great-spheres are dual to the vertices of a neighborly polytope. Equivalently, all subarrangements of dimension $p \ge d/2$ have bi-polar depth functions. We generalize the face-counting formulas for neighborly polytopes to the levels in neighborly arrangements. In particular, we show that the number of *p*-cells at depth *k* is a function of *n*, *d*, *p*, and *k* alone. For the special case of cyclic polytopes, this was proved before by Andrezejak and Welzl (2003, Theorem 5.1), who also derived explicit formulas for the number of cells.

### Equations in matrix form

We write $d = 2t-1$ for odd *d* and $d = 2t$ for even *d*. Let ${\mathcal {A}}$ be a neighborly arrangement of *n* great-spheres in ${\mathbb {S}}^d$, so all subarrangements of dimension $t \le p \le d$ are bi-polar. By Theorem 1, the $E_{k}^{p}$ are simple functions in *n*, *d*, *p*, and *k*, for all $t \le p \le d$. In addition, we get *t* independent relations for every *k* from Theorem 2. Specifically, for every odd *p* between 0 and *d*, we get a relation by equating the left-hand side of (1) with the right-hand side of (1). This gives what we call a *giant linear system* with variables $E_{k}^{0}$ to $E_{k}^{t-1}$ for $0 \le k \le n$. To describe it, we introduce the $t \times t$ matrices $M_d$. For odd *d*, it is a straightforward configuration of binomial coefficients, which is however interrupted by $-2$s replacing $$- \left( {\begin{array}{c}2t-j\\ 2i-2\end{array}}\right) = -1$$ in row *i* and column *j* whenever $2t-j = 2i-2$:

35 $$\begin{aligned} M_{2t-1}&= \left[ \begin{array}{llllll} \genfrac(){0.0pt}1{2t-1}{0} &{} -\genfrac(){0.0pt}1{2t-2}{0} &{} \genfrac(){0.0pt}1{2t-3}{0} &{} -\genfrac(){0.0pt}1{2t-4}{0} &{} \ldots &{} \pm \genfrac(){0.0pt}1{t}{0} \\ \genfrac(){0.0pt}1{2t-1}{2} &{} -\genfrac(){0.0pt}1{2t-2}{2} &{} \genfrac(){0.0pt}1{2t-3}{2} &{} -\genfrac(){0.0pt}1{2t-4}{0} &{} \ldots &{} \pm \genfrac(){0.0pt}1{t}{2} \\ \vdots &{} \vdots &{} \vdots &{} \vdots &{} \ddots &{} \vdots \\ \genfrac(){0.0pt}1{2t-1}{2t-4} &{} -\genfrac(){0.0pt}1{2t-2}{2t-4} &{} \genfrac(){0.0pt}1{2t-3}{2t-4} &{} -2 &{} \ldots &{} 0 \\ \genfrac(){0.0pt}1{2t-1}{2t-2} &{} -2 &{} 0 &{} 0 &{} \ldots &{} 0 \end{array} \right] . \end{aligned}$$

These replacements will be important shortly. For even *d*, the matrix $M_{2t}$ has the same number of entries, with $$\left( {\begin{array}{c}2t-j+1\\ 2i-1\end{array}}\right) $$ in row *i* and column *j* replacing $$\left( {\begin{array}{c}2t-j\\ 2i-2\end{array}}\right) $$ in $M_{2t-1}$. The $-2$s and 0s are the same in both matrices. In *d* dimensions, the giant system is given by a $t(n+1) \times t(n+1)$ matrix, with $n+1$ copies of $M_d$ along the diagonal. All entries to the lower left of this diagonal of $t \times t$ blocks are zero, while there are sporadic non-zero entries to the upper right.

#### Lemma 6

(Invertible blocks imply invertible systems) For every $d \ge 1$, if $M_d$ is invertible, then the giant system of linear relations in *d* dimensions is invertible.

#### Proof

If $M_d$ is invertible, then we can use row and column operations to turn $M_d$ into an upper triangular matrix with non-zero entries along the diagonal. Applying the same operations to the giant matrix, we get a giant upper triangular matrix with non-zero entries along the entire diagonal. $\square$

### Everything modulo 2

We prove the invertibility of $M_{2t-1}$ by proving that its determinant is odd. Equivalently, we write $P_{2t-1}$ for the matrix $M_{2t-1}$ in which every entry is replaced by its parity, and we show that the mod 2 determinant of $P_{2t-1}$ is 1. Before doing so, we show that the invertibility of $M_{2t-1}$ implies the invertibility of $M_{2t}$. Let $N_{2t}$ be the matrix $M_{2t}$ after dividing each column by the largest power of 2 that divides all its entries, and write $P_{2t}$ for the matrix $N_{2t}$ in which every entry is replaced by its parity.

#### Lemma 7

(Odd imply even invertible blocks) $P_{2t} = P_{2t-1}$.

#### Proof

Recall that the entry in row *i* and column *j* is $$\left( {\begin{array}{c}2t-j\\ 2i-2\end{array}}\right) $$ in $M_{2t-1}$ and $$\left( {\begin{array}{c}2t-j+1\\ 2i-1\end{array}}\right) $$ in $M_{2t}$, unless this entry is $-2$ or 0, in which case it is the same in the two matrices. Assuming the former case, the ratio of the two entries is $$\left( {\begin{array}{c}2t-j+1\\ 2i-1\end{array}}\right) / \left( {\begin{array}{c}2t-j\\ 2i-2\end{array}}\right) = (2t-j+1)/(2i-1)$$. Since $2i-1$ is odd, the largest power of 2 that divides $$\left( {\begin{array}{c}2t-j+1\\ 2i-1\end{array}}\right) $$ is the largest power of 2 that divides $$\left( {\begin{array}{c}2t-j\\ 2i-2\end{array}}\right) $$ times the largest power of 2 that divides $2t-j+1$. The latter is the same for all entries in a column. We thus divide column *j* in $M_{2t}$ by the largest power of 2 that divides $2t-j+1$, which is 1 for all even *j*. The even columns of $M_{2t}$ are the ones that contain the $-2$s, so after dividing, the parities of corresponding terms in $M_{2t}$ and $M_{2t-1}$ are the same. Equivalently, $P_{2t} = P_{2t-1}$. $\square$

Henceforth, we focus on the odd case. We use a consequence of Kummer’s theorem (Kummer 1852) to get the parity version of $M_{2t-1}$:

#### Lemma 8

(Odd binomial coefficients) For all $0 \le k \le n$, $$\left( {\begin{array}{c}n\\ k\end{array}}\right) $$ is odd iff the binary representations of *n*, *k*, and $n-k$ satisfy $n_2 = k_2 \mathtt{~xor~} (n-k)_2$.

**Fig. 3:**
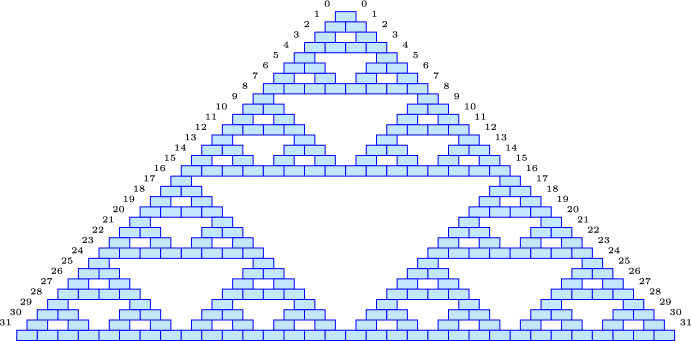
The Pascal triangle in modulo 2: the *blue* bricks are odd entries, and the *white* bricks (not shown) are even entries (colour figure online)

In words: the 1s in the binary representations of *k* and $n-k$ are at disjoint positions. It follows that the positions of the 1s in the binary representation of *k* are a subset of the positions of the 1s in the binary representation of *n*, and similarly for $n-k$ and *n*. A compelling visualization of Lemma 8 is the Pascal triangle in binary, whose 1s form the Sierpinski gasket as shown in Fig. 3.

**Fig. 4:**
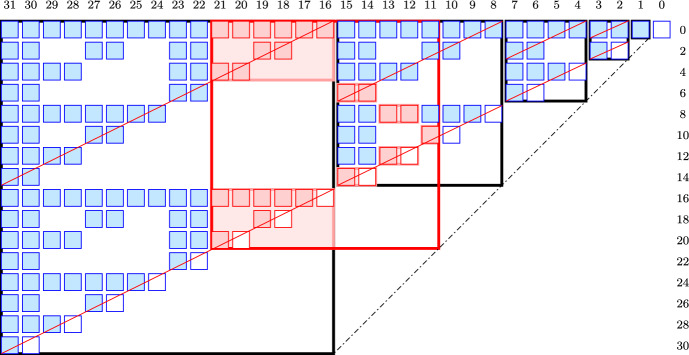
Each *blue* and *pink* square is a 1 in the matrix, and each *white* square is a 0 (only those originally equal to $-2$ are shown). The *bold black* frames mark the exponential blocks, the *bold red* frame marks the 11-th block, $P_{21}$, and the *pink* boxes inside the *red* frame mark the tops and bottoms of the NE- and SW-incursions that arise in its reduction (colour figure online)

To transform the Sierpinski gasket into a matrix that contains $P_{2t-1}$, for every $t \ge 1$, we drop every other up-slope (whose label, given along the down-slope in Fig. 3, is odd), we draw the remaining up-slopes as rows, and we draw the horizontal lines in the gasket as columns. Finally, we convert the last 1 in each row to a 0. These are the binomial coefficients that change from $-1$ to $-2$ in $M_{2t-1}$; see Fig. 4.

### Reducing exponential blocks

Observe that $P_{2t-1}$ is the submatrix consisting of the rows labeled 2*i*, for $0 \le i \le t-1$, and the columns labeled *j*, for $t \le j \le 2t-1$; see Fig. 4. We call this the *t*-*th block*. For the time being, we focus on *exponential blocks*, for which *t* is a power of 2. Note the symmetry between the upper and lower halves of an exponential block: the bottom is a copy of the top, except that the last 1 in each row is turned into a 0. We use this property to reduce exponential blocks.

#### Reduction 1

(Exponential block) Let $P_{2t-1}$ be an exponential block, with $t = 2^n$, and write $s = 2^{n-1}$. We reduce $P_{2t-1}$ in three steps:

1. For $0 \le i \le s-1$, add the row with label $2i+2s$ to the row with label 2*i*. Thereafter, we have a 1 in each row and each even column, and otherwise only 0s in the upper half of the exponential block.
2. Zero out the even columns in the lower half using the rows in the upper half. After consolidating the lower half by removing the even columns, which are all zero, we get an upper triangular matrix with 1s in the diagonal.
3. Reduce this upper triangular matrix to the $s \times s$ identity matrix. Adding the even columns back, we have a 1 in each row and each odd column, and otherwise only 0s in the lower half of the exponential block.

Assuming $t = 2^n$, the above reduction algorithm turns $P_{2t-1}$ into a $t \times t$ permutation matrix, whose determinant is of course 1. This is the parity of the determinant of $M_{2t-1}$, which is therefore non-zero. To extend this result to integers, *t*, that are not necessarily powers of 2, we need a few properties of an exponential block. Being a square matrix with $t = 2^n$ rows and columns, it decomposes into four quarters of $s = 2^{n-1}$ rows and columns each. By combining the NE- and NW-quarters, we get the *northern half* of the exponential block, and we draw the line from its bottom-left to top-right corners, calling it the *northern diagonal*; see Fig. 4. Similarly, we merge the SE- and SW-quarters to get the *southern half* and draw the *southern diagonal* from the bottom-left to top-right corner. Note that the southern half of $P_{2t-1}$ is a copy of everything to the right of the northern half, namely the exponential blocks of size $1, 2, 4, \ldots , 2^{n-1}$ plus the 0s below and to the right of them.

An *NE-incursion* is a submatrix whose bottom-left corner lies on the southern diagonal and whose top-right corner is the top-right corner of the exponential block. As an example consider the rows labeled 0 to 20 and columns labeled 21 to 16, which is an NE-incursion of $P_{31}$ in Fig. 4. We decompose the NE-incursion into three rectangular matrices stacked on top of each other: the *top*, the *middle*, and the *bottom*, in which the top and bottom are twice as wide as they are high, and the middle fills the space in between. Importantly, the middle is zero, and the top and bottom combine to a square matrix whose structure is such that Reduction  1 can reduce it to the identity matrix.

Symmetrically, an *SW-incursion* is a submatrix whose top-right corner lies on the northern diagonal and whose bottom-left corner is the bottom-left corner of the exponential block. As an example consider the rows labeled 6 to 14 and columns labeled 15 to 14, which is an SW-incursion of $P_{15}$ in Fig. 4. As before, we decompose the SW-incursion into three rectangular matrices, in which the *top* and *bottom* are twice as wide as they are high, and the *middle* consists of the remaining rows in between. The top and bottom combine again to a square matrix that can be reduced to the identity matrix by Reduction 1. However, the middle is not necessarily zero. On the other hand, all entries to the right of the top but still within the exponential block are zero.

### Reducing general blocks

We thus have the necessary ingredients to reduce a not necessarily exponential block, $P_{2t-1}$. Assuming *t* is not a power of 2, let *u* be the power of 2 such that $u/2< t < u$, and write $s = u/2$. The overlap of $P_{2t-1}$ with $P_{2u-1}$ is an NE-incursion of the latter.

#### Reduction 2

(NE-incursion) Let *I* be the overlap of $P_{2t-1}$ and $P_{2u-1}$. We reduce *I* and zero out portions of $P_{2t-1}$ outside *I*:

1. Combine the top and bottom of *I* and reduce it using Reduction 1.
2. Add back the middle, which we recall is 0.
3. Use the columns of the reduced *I* to zero out the rectangular regions of $P_{2t-1}$ to the right of the top and bottom of *I*.

Step 1 may contaminate the regions to the right of the bottom of *I* with non-zero entries, but Step 3 cleans up the contamination at the end. We are thus left with an un-reduced submatrix of size $(u-t) \times (u-t)$, which we denote $P_{2t-1}'$. It is a bottom-left submatrix but not necessarily an SW-incursion of $P_{2s-1}$. Assuming $s < 2(u-t)$, there is a largest SW-incursion of $P_{2s-1}$ contained in $P_{2t-1}'$, which has the same number of rows as $P_{2t-1}'$.

#### Reduction 3

(SW-incursion) Assume $s < 2(u-t)$ and let *J* be the largest SW-incursion of $P_{2s-1}$ contained in $P_{2t-1}'$. We reduce *J* as follows:

1. Combine the top and bottom of *J* and reduce it using Reduction 1.
2. Add back the middle and zero it out using row operations.

We note that the regions of $P_{2t-1}'$ to the right of the top and bottom of *J* are zero because *J* is an SW-incursion, and $P_{2t-1}'$ is contained in $P_{2s-1}$. Step 1 preserves this property, so Step 2 can zero out the middle without contaminating the remaining un-reduced matrix of size $(s-u+t) \times (s-u+t)$, which we denote $P_{2t-1}''$.

It is also possible that $s \ge 2(u-t)$, in which case there is no non-empty SW-incursion of $P_{2s-1}$ contained in $P_{2t-1}'$. We thus substitute the SW-quarter of $P_{2s-1}$ for $P_{2s-1}$, or the SW-quarter of that SW-quarter, etc. This square matrix is a copy of the exponential block of the same size, so Reduction 3 still applies. Similarly, $P_{2t-1}''$ is a copy of the $(s-u+t)$-th block. Since $s-u+t < t$, we can reduce it by induction. The correctness of the reduction algorithms implies

#### Lemma 9

(Blocks are invertible) For every $d \ge 1$, $M_d$ is invertible.

#### Proof

For $d = 2t-1$, Reductions 1–3 together with induction imply that $P_{2t-1}$ can be reduced to the identity matrix. By Lemma 7 this is also the case for $P_{2t}$. Since $P_d$ is the parity version of $M_d$, this implies that $M_d$ is invertible. $\square$

### Number of cells

The invertibility of the blocks implies the invertibility of the giant linear systems, which implies that the number of cells in the levels of neighborly arrangements are independent of the geometry of the great-spheres defining the arrangement.

#### Theorem 3

(Neighborly arrangements) Let ${\mathcal {A}}$ be a neighborly arrangement of $n \ge d$ great-spheres in ${\mathbb {S}}^d$. Then the $E_{k}^{p} ({\mathcal {A}})$ and the ${C}_{k}^{p} ({\mathcal {A}})$ are functions of *n*, *d*, *p*, and *k*.

#### Proof

By Lemma 9, the matrix $M_d$ is invertible, which by Lemma 6 implies that the giant linear system created from Theorems 1 and 2 is invertible. Hence, the $E_{k}^{p} ({\mathcal {A}})$ of the *d*-dimensional arrangement are determined; that is: they are functions of *n*, *d*, *p*, and *k*, but not of the great-spheres defining the arrangement. By Theorem 2, the ${C}_{k}^{p} ({\mathcal {A}})$ are determined by the $E_{k}^{p} ({\mathcal {A}})$, so they are also functions of *n*, *d*, *p*, and *k*. $\square$

As an example, consider a neighborly arrangement of *n* great-spheres in ${\mathbb {S}}^4$. All subarrangements of dimension 2, 3, and 4 have bi-polar depth functions, so we get the $E_{k}^{p}$ for $p = 2,3,4$ from Theorem 1, and we use Theorem 2 to get them for $p = 0,1$:

36 $$\begin{aligned} E_{k}^{0}&= \tfrac{1}{2} (k+1) n (n-k-3) \quad \text{ for } \quad 0 \le k \le n-4, \end{aligned}$$

37 $$\begin{aligned} E_{k}^{1}&= n (n-2k-3) \quad \text{ for } \quad 0 \le k \le n-3, \end{aligned}$$

38 $$\begin{aligned} E_{k}^{2}&= \genfrac(){0.0pt}1{n}{2}, ~0, ~\genfrac(){0.0pt}1{n}{2} \quad \text{ for } k=0, \quad 1 \le k \le n-3, \quad k=n-2 , \end{aligned}$$

39 $$\begin{aligned} E_{k}^{3}&= n, ~0, ~-n \quad \text{ for } \quad k=0,\quad 1 \le k \le n-2,\quad k=n-1, \end{aligned}$$

40 $$\begin{aligned} E_{k}^{4}&= 1, ~0, ~1 \quad \text{ for } \quad k=0,\quad 1 \le k \le n-1,\quad k=n. \end{aligned}$$

Using the relations ${C}_{k}^{0} = E_{k}^{0}$, ${C}_{k}^{1} = 4 E_{k}^{0} - E_{k}^{1}$, etc., from Theorem 2, we get the number of cells with given depth:

41 $$\begin{aligned} {C}_{k}^{0}&= \tfrac{1}{2} (k+1) n (n-k-3) \quad \text{ for } \quad 0 \le k \le n-4, \end{aligned}$$

42 $$\begin{aligned} {C}_{k}^{1}&= n [n(2k+1) - 2k^2 - 6k -3] \quad \text{ for } \quad 0 \le k \le n-3, \end{aligned}$$

43 $$\begin{aligned} {C}_{k}^{2}&= \genfrac(){0.0pt}1{n}{2}, ~3nk (n-k-2), ~\genfrac(){0.0pt}1{n}{2} \quad \text{ for } k=0, \quad 1 \le k \le n-3,\quad k=n-2 , \end{aligned}$$

44 $$\begin{aligned} {C}_{k}^{3}&= n, ~n[(2k-1)n-2k^2-2k+3], ~6\genfrac(){0.0pt}1{n}{2}, ~2\genfrac(){0.0pt}1{n}{2}, ~n \nonumber \\&\text{ for } k=0,\quad 1 \le k \le n-4, \quad k=n-3, \quad k=n-2,\quad k=n-1, \end{aligned}$$

45 $$\begin{aligned} {C}_{k}^{4}&= 1, ~\tfrac{1}{2} n [n(k-1)-k^2+3], ~n(n-3), ~\genfrac(){0.0pt}1{n}{2} , ~n , ~1 \nonumber \\&\text{ for } k=0,\quad 1 \le k \le n-4,\quad k=n-3,\quad k=n-2,\quad k=n-1,\quad k=n. \end{aligned}$$

## Discussion

The main contribution of this paper is the introduction of the discrete depth function as a topological framework to approach questions in discrete geometry, and the establishment of the system of Dehn–Sommerville–Euler relations for levels of this function. We have illustrated the use of this system by rederiving known cell-counting formulas for order-*k* Voronoi tessellations in ${\mathbb {R}}^2$ and ${\mathbb {R}}^3$, and by extending the classic face-counting formulas for neighborly polytopes to the levels in neighborly arrangements. This work suggests further research to deepen our understanding of the framework:

- Establish effective relations expressing the connections between the restrictions of the depth function to subarrangements.
- Relate the stability of the persistence diagrams of restrictions of the depth function to combinatorial questions in geometry.

While our framework sheds new light on well studied questions in discrete geometry, there is plenty of work that remains. The following questions are of particular interest:

- Give bounds on the topological quantities that arise in counting the regions of order-*k* Voronoi tessellations. As established in Biswas et al. (2021), the relevant quantity in ${\mathbb {R}}^3$ is the double sum of depth characteristics of the 2-dimensional cells (the polygons) in the corresponding arrangement of great-spheres in ${\mathbb {S}}^4$. How do these results extend beyond 3 dimensions?
- Generalize the results on neighborly arrangements to counting the *k*-sets of general sets of *n* points in ${\mathbb {R}}^d$. Specifically, use the framework of depth functions to improve the current best upper bounds on the maximum number of *k*-sets, which are $O(n^{4/3})$ in ${\mathbb {R}}^2$ (Dey 1998), $O(n^{5/2})$ in ${\mathbb {R}}^3$ (Sharir et al. 2001), and $O(n^{d-\varepsilon _d})$ for a small constant $\epsilon _d > 0$ in ${\mathbb {R}}^d$ (Živaljević and Vrećica 1992).
